# GABA Signaling and Neuroactive Steroids in Adrenal Medullary Chromaffin Cells

**DOI:** 10.3389/fncel.2016.00100

**Published:** 2016-04-18

**Authors:** Keita Harada, Hidetada Matsuoka, Hiroaki Fujihara, Yoichi Ueta, Yuchio Yanagawa, Masumi Inoue

**Affiliations:** ^1^Department of Cell and Systems Physiology, University of Occupational and Environmental Health School of MedicineKitakyushu, Japan; ^2^Department of Physiology, University of Occupational and Environmental Health School of MedicineKitakyushu, Japan; ^3^Department of Genetic and Behavioral Neuroscience, Gunma University Graduate School of MedicineMaebashi, Japan

**Keywords:** GABA, adrenal chromaffin cell, PC12 cell, neuroactive steroid, paracrine, GAD67, GABA_A_ receptors, immature subunit

## Abstract

Gamma-aminobutyric acid (GABA) is produced not only in the brain, but also in endocrine cells by the two isoforms of glutamic acid decarboxylase (GAD), GAD65 and GAD67. In rat adrenal medullary chromaffin cells only GAD67 is expressed, and GABA is stored in large dense core vesicles (LDCVs), but not synaptic-like microvesicles (SLMVs). The α3β2/3γ2 complex represents the majority of GABA_A_ receptors expressed in rat and guinea pig chromaffin cells, whereas PC12 cells, an immortalized rat chromaffin cell line, express the α1 subunit as well as the α3. The expression of α3, but not α1, in PC12 cells is enhanced by glucocorticoid activity, which may be mediated by both the mineralocorticoid receptor (MR) and the glucocorticoid receptor (GR). GABA has two actions mediated by GABA_A_ receptors in chromaffin cells: it induces catecholamine secretion by itself and produces an inhibition of synaptically evoked secretion by a shunt effect. Allopregnanolone, a neuroactive steroid which is secreted from the adrenal cortex, produces a marked facilitation of GABA_A_ receptor channel activity. Since there are no GABAergic nerve fibers in the adrenal medulla, GABA may function as a para/autocrine factor in the chromaffin cells. This function of GABA may be facilitated by expression of the immature isoforms of GAD and GABA_A_ receptors and the lack of expression of plasma membrane GABA transporters (GATs). In this review, we will consider how the para/autocrine function of GABA is achieved, focusing on the structural and molecular mechanisms for GABA signaling.

GABA is synthesized not only in the brain, but also in peripheral tissues and organs, such as the pancreas (Okada et al., 1976) and the adrenal medulla. The presence of GABA in the bovine adrenal medulla was first reported in 1984 (Kataoka et al., 1984), and thereafter GABA’s actions in adrenal medullary chromaffin cells of dogs (Kataoka et al., 1986), cattle (Peters et al., 1989), rats (Matsuoka et al., 2008), and guinea pigs (Inoue et al., 2013) *in vivo* and/or *in vitro* have been functionally explored.

Compared to the adrenal medulla, there is ample research on GABA in the brain where it is the major inhibitory neurotransmitter. This review is focused on the functions of GABA in the adrenal chromaffin cells, but a brief recapitulation of GABA signaling in the brain will help our understanding of GABA functions in the adrenal medulla. Neuronal GABA is synthesized from glutamic acid by glutamic acid decarboxylase (GAD), an enzyme that is encoded by two different genes, *GAD2* (GAD65) and *GAD1* (GAD67; Obata, 2013). GAD exists as a homo- or heterodimer of GAD65 and GAD67. GAD65 is mainly present in the nerve terminal, whereas GAD67 is diffusely distributed in the cell body as well as nerve terminals (Pinal and Tobin, 1998; Soghomonian and Martin, 1998; Obata, 2013). GAD67 plays the major role for GABA production in the embryonic brain (Asada et al., 1997), whereas the contribution of GAD65 begins to increase after birth. In the adult 50–70% of the total GABA in the brain is produced by GAD65 and the remaining GABA by GAD67 (Stork et al., 2000). Thus, both isoforms are involved in production of GABA in adult brain (Pinal and Tobin, 1998; Obata, 2013). The development of GAD65 appears to parallel that of inhibitory neuronal transmission in the brain (Greif et al., 1992). The subtypes of GABA_A_ receptors also change during the brain development (Laurie et al., 1992; Ortinski et al., 2004). These results suggest that the function of GABA in the embryonic brain differs from its role as a neurotransmitter in the mature brain (Ben-Ari et al., 2007; Pallotto and Deprez, 2014). Thus, it is likely that GABAergic transmission has multiple functions which may change during brain development. However, how the expression of GABA signaling molecules coordinately changes remains to be fully elucidated (Succol et al., 2012).

In this review, we will consider the functions of GABA in the adrenal medulla while referring to GABA signaling in neurons and other endocrine cells. Special focus will be placed on the structural and molecular mechanisms for GABA actions in chromaffin cells. Overall the data indicate that the expression of GABA signaling molecules in adrenal chromaffin cells is finely tuned for GABA to function as a para/autocrine factor to modulate catecholamine secretion.

## Morphology of GABA Signaling in the Adrenal Medulla

### Localization of GABA

**Figure 1:**
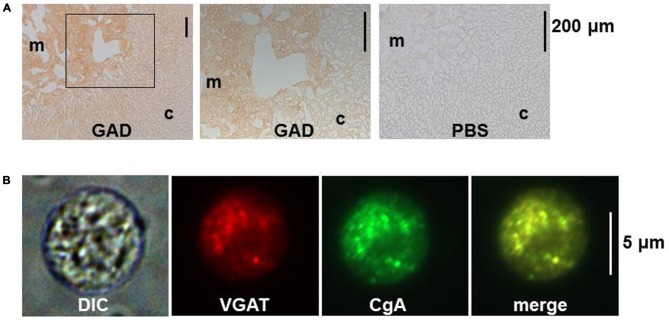
**Immunostaining for glutamic acid decarboxylase (GAD) and vesicular GABA transporter (VGAT) in rat adrenal medullary chromaffin cells. (A)** Immunohistochemistry for GAD in rat adrenal gland. Sections were incubated overnight in PBS with (GAD) or without (PBS) rabbit anti-GAD antibody (Ab). The bound Ab was detected with the indirect immunoperoxidase method. The middle panel represents an enlargement of the area indicated by the square in the left panel. **(B)** Immunocytochemistry for VGAT in a dissociated rat adrenal medullary cell. The adrenal medulla was treated with collagenase and then cells were mechanically dissociated with needles. The cells were treated overnight with rabbit anti-VGAT Ab and goat anti-chromogranin A (CgA) Ab and then with secondary Abs conjugated with Alexa 488 and Alexa 546. Images from left to right represent differential interference contrast (DIC), rhodamine-like, FITC-like, and merge of the second and the third. **(A,B)** Are reproduced from Matsuoka et al. (2008).

When the localization of GABA was examined in bovine, dog and mouse adrenal medullae with immune-labeling approaches, GAD- and/or GABA-like immunoreactivities (IRs) were detected in chromaffin cells and nerve fiber-like structures (Kataoka et al., 1984, 1986; Iwasa et al., 1998). Previously, retrograde tracing had revealed that several areas in the brain and the spinal cord had materials that were retrogradely transported from the rat adrenal medulla (Mohamed et al., 1988; Coupland et al., 1989). Thus, it is possible that neurons in such areas send GABAergic nerve fibers to adrenal chromaffin cells. However, this idea has not been supported by pharmacological analyses of neuronal transmission. Fast synaptic currents evoked in slices of the rat adrenal medulla by field stimulation were completely suppressed by application of hexamethonium, a nicotinic ACh receptor blocker (Barbara and Takeda, 1996; Kajiwara et al., 1997). These electrophysiological findings would negate the possible innervation by GABAergic fibers in the adrenal medulla. This notion is further supported by immunostaining for GAD and vesicular GABA transporter (VGAT), which is involved in the uptake of GABA into vesicles. GAD proteins were recognized in the rat adrenal medulla by immunoblotting, and GAD-like IR was present in rat adrenal chromaffin cells but not in fiber-like structures (Figure 1). Furthermore, the mRNA for GAD67, but not GAD65, was detected in rat adrenal medullae by RT-PCR analysis (Matsuoka et al., 2008; Inoue et al., 2010), suggesting that the GAD activity was not in nerve terminals. VGAT was also detected in rat adrenal chromaffin cells at the mRNA and protein levels (Matsuoka et al., 2008). The immunostaining for GAD and VGAT unambiguously indicate that GABA is synthesized in chromaffin cells, but not nerve fibers, in the adrenal medulla of at least rats.

### GABA-Storing Secretory Vesicles

Chromaffin cells have two kinds of secretory vesicles: one is the large dense core vesicles (LDCVs) or chromaffin granules with a mean diameter of 356 nm (Plattner et al., 1997), where catecholamines are stored together with chromogranins and ATP. The second is synaptic-like microvesicles (SLMVs) with a mean diameter of 90 nm (Plattner et al., 1997), which correspond to synaptic vesicles in the nerve terminal (Thomas-Reetz and De Camilli, 1994). There is morphological and biochemical evidence that GABA is stored in LDCVs, but not in SLMVs, in rat and bovine adrenal chromaffin cells (Inoue et al., 2010). First, VGAT was co-localized with chromogranin, a marker protein of LDCVs (Figure 1B), but not synaptophysin, an integral protein of SLMVs (Navone et al., 1986). Also, fractionation analysis of homogenates of the bovine adrenal medulla revealed that VGAT was detected in the fractions of high density, where dopamine β-hydroxylase, an enzyme involved in the synthesis of noradrenaline from dopamine, was enriched, whereas synaptophysin was enriched in fractions of intermediate density (Harada et al., 2010). When the homogenate was centrifuged and divided into supernatent and pellet fractions and then the pellet was subjected to sucrose density fractionation, GABA was recovered together with catecholamines in high density fractions. Furthermore, when a fusion protein of green fluorescent protein with VGAT (GFP-VGAT) was exogenously expressed in the PC12 cell, an immortalized cell line of rat adrenal chromaffin cells, it was localized in organelles immunopositive for chromogranin. In contrast, a GFP-vesicular acetylcholine transporter fusion protein (GFP-VAChT) was mainly present in organelles that were immunopositive for synaptophysin, suggesting that GFP-VGAT and GFP-VAChT were located in LDCVs and SLMVs, respectively (Harada et al., 2010).

The two kinds of secretory vesicles also occur in pancreatic β cells (Reetz et al., 1991) where insulin and GABA are produced. Insulin is stored in LDCVs. In contrast to chromaffin cells, GABA is stored in both SLMVs and in LDCVs in β cells of at least rats (Reetz et al., 1991; Gammelsaeter et al., 2004): gold particles for GABA and VGAT were concentrated much more in SLMVs rather than in LDCVs.

### Functions of LDCVs and SLMVs

Whether GABA is stored in LDCV or SLMV is important for its physiological role. Secretion mediated by SLMVs occurs rapidly compared with that of LDCVs (Kasai, 1999), for example in PC12 cells the time constants for exocytosis differ by 10× or more between SLMVs and LDCVs. This difference between SLMVs and LDCVs may be accounted for by the synaptotagmin isoforms mainly present in these two vesicles. The Ca^2+^ sensitivity and kinetics of Ca^2+^ interaction of synptotagmin differ among isoforms: Ca^2+^ affinity of synaptotagmin 7 is much higher than that of synaptotagmin 1 (Wang et al., 2005), and the kinetics of the former is slower than that of the latter (Hui et al., 2005). Thus, the presence of synptotagmin 1 as the main isoform in synaptic vesicles in the nerve terminal (Geppert et al., 1994) would allow action potentials arriving in the nerve terminal to trigger a rapid secretion of neurotransmitters, so that neurotransmission can occur with a delay of less than 1 ms (Johnston and Wu, 1995; Minneci et al., 2011). As in other secretory cells, the main isoforms of synaptotagmins present in LDCV and SLMV in chromaffin cells are different. The major isoform present in LDCVs is synptotagmin 7, whereas that in SLMVs is synptotagmin 1 although synaptotagmin 1 is found in LDCV (Matsuoka et al., 2011).

As mentioned previously, a part of the GABA molecules in pancreatic β cells are likely stored in SLMVs, and GABA has been shown to inhibit glucagon secretion from pancreatic α cells (Rorsman et al., 1989; Xu et al., 2006). This GABA-mediated inhibition of glucagon secretion may occur under conditions where β cells are strongly excited, so that synaptotagmin 1 in SLMVs is activated with the consequent release of GABA. That is, in the islets of Langerhans GABA released from β cells may act in a paracrine manner to reduce glucagon release from α cells. In contrast to β cells, in chromaffin cells GABA is stored in LDCV rather than SLMV. The storage of GABA in LDCVs suggests that GABA plays a modulatory role for secretion of catecholamines. As the amount of secreted catecholamine increases, that of GABA also increases and so acts to reduce high levels of secretion (see “Dual Action of GABA” Section below).

## GABA_A_ Receptors in Chromaffin Cells

Receptors for GABA comprise ionotropic GABA_A_ receptors containing a directly-gated ion channel and metabotropic GABA_B_ receptors (Inoue et al., 1985). Bovine chromaffin cells in culture have been shown to express GABA_B_ receptors by functional analyses (Castro et al., 1989). However, rat chromaffin cells failed to respond to baclofen, a GABA_B_ receptor agonist, and GABA-induced currents were suppressed by bicuculline, a GABA_A_ receptor antagonist (Matsuoka et al., 2008), indicating the expression of GABA_A_ receptors, but not GABA_B_ receptors. Therefore, only GABA_A_ receptors will be discussed here.

### Subunit Composition of GABA_A_ Receptors

GABA_A_ receptors are a pentamer of α (α1 to α6) and β (β1 to β3) subunits, with one or more γ (γ1 to γ3), ρ (ρ1 to ρ3), δ, ɛ, or θ subunits. The possible combinations of these subunits are enormous, but the actual isoforms of GABA_A_ receptors present in the brain are limited (Rudolph and Knoflach, 2011): 60% of them are the complex of two α1, two β, and one γ2 subunits, and in 20% of GABA_A_ receptors, two molecules of the α2, α3, or α5 subtype are combined with two β and one γ2 subunits. In the brain, GABA_A_ receptor expression begins early in development (Serafini et al., 1998; Ben-Ari et al., 2007), and their subunit combination changes during development (Laurie et al., 1992; Ortinski et al., 2004). The α2 and α3 subunits are predominantly expressed in the embryonic rat brain, while the α1 subunit becomes predominant during the first 3 weeks of postnatal life (Okada et al., 2000; Bosman et al., 2002; Ehrlich et al., 2013). Because of this developmental change, α2 and α3 are called immature subunits, whereas α1 is a mature subunit. The replacement of α2 or α3 subunits with α1 results in a shortening of the time course of the decay of inhibitory postsynaptic currents, probably because the open time of α1-containing GABA_A_ receptors is shorter than that of α3-containing receptors (Mozrzymas et al., 2007). Thus, this replacement leads to increased temporal precision of inhibitory neurotransmission in the brain. It has been suggested that in the embryonic brain GABAergic transmission functions in a more general, volume transmission mode that converts to a more precise, synaptic mode with development (Represa and Ben-Ari, 2005).

In contrast to the mature brain, GABA in the adrenal medulla is not involved in synaptic neurotransmission. The combination of subunits in chromaffin cells may reflect the physiological function which GABA mediates. RT-PCR and immunoblotting of rat adrenal medullae revealed the expression of α1 and α3 subunits (Matsuoka et al., 2008). With immunoprecipitation these subunits were confirmed to be complexed with β2/3 and γ2 or δ subunits. However, a recent quantitative analysis of protein expression revealed that the α3 subunit is the major α subunit expressed in adrenal medullae of not only rats, but also guinea pigs (Inoue et al., 2013). The enhancement of GABA-induced currents by L-838, 417, an α3-selective benzodiazepine analog, in guinea-pig chromaffin cells supports the dominant expression of α3 and indicates that the α3 subunit is mainly complexed with γ2 (Inoue et al., 2013). Furthermore, GABA-induced currents were suppressed by Zn^2+^ in a dose-dependent manner and the inhibition was well described by a rectangular hyperbola, suggesting that GABA_A_ receptors in chromaffin cells are homogeneous (Inoue et al., 2013). To summarize, GABA_A_ receptors in chromaffin cells are mainly represented by the α3β2/3γ2 complex.

### Regulation of GABA_A_ Receptor Expression

**Figure 2:**
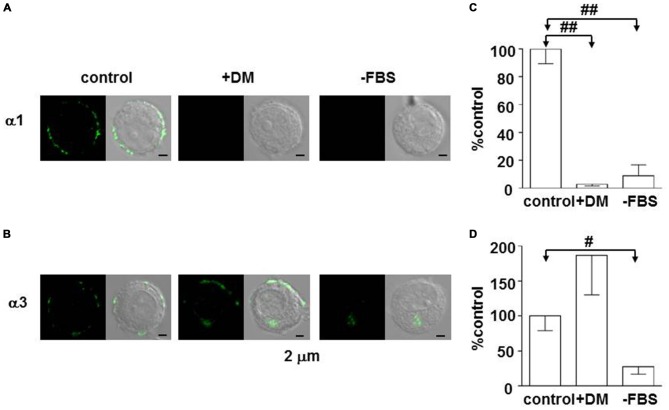
**Enhancement of expression of the GABA_A_ α3 subunit in PC12 cells by dexamethasone. (A,B)** Immunofluorescent staining for α1 and α3 subunits in PC12 cells, respectively. Cells were cultured for 1 week in Dulbecco’s modified medium supplemented with 10% fetal bovine serum (FBS; control), Dulbecco’s modified medium with 10 μM dexamethasone (+DM), or Dulbecco’s modified medium (−FBS). Left and right images represent confocal fluorescent images and merge of fluorescent and DIC images. **(C,D)** Summaries of α1- and α3-like immunoreactivities, respectively. The amounts of α1- and α3-like immunoreactivities were measured by using ImageJ software (NIH, Bethesda, MD, USA). The amounts of the immunoreactivities in PC12 cells cultured with DM (+DM) and without DM and FBS (−FBS) were expressed as percentages of those in the control PC12 cells, which were measured under the same conditions. The data indicate mean ± SEM (**C**, *n* = 12, 12, and 10 for control, +DM, and −FBS, respectively; **D**, *n* = 19, 9, and 10 for control, +DM, and −FBS, respectively). ^#^and ^##^represent statistical significance of *P* < 0.01 and *P* < 0.001, respectively. **(B,D)** Are reproduced from Inoue et al. (2013).

The fact that the α3β2/3γ2 complex constitutes the majority of GABA_A_ receptors means chromaffin cells continue to express immature GABA_A_ receptors in adulthood, which raises a question about how the control of subunit expression may differ between adrenal gland and brain. The internal environment drastically changes in the growth process from the prenatal period to the neonate and then juvenile periods. One such change in rats is a decrease in blood glucocorticoid during the first 2 weeks of life (Sapolsky and Meaney, 1986). Early life experience, including impaired maternal care, influences brain development and the expression of GABA_A_ receptors in the adulthood (Caldji et al., 1998, 2003), and the blood glucocorticoid concentration increases in rats subjected to maternal deprivation (Chen et al., 2012). These observations suggest that glucocorticoids play an important role for regulation of GABA_A_ receptors. To examine the effects of glucocorticoids on the expression of GABA_A_ receptors, PC12 cells were used. PC12 cells were found to express α1 as well as α3 subunits, which were mainly present at the cell periphery (Inoue et al., 2013). The expression of both subunits was abolished by removal of serum from the culture medium, but that of α3—but not α1—was restored by addition of the synthetic glucocorticoid dexamethasone (Figure 2), indicating that glucocorticoid activity supports α3 expression. There are two kinds of receptors in the cytosol to which glucocorticoids bind: one is mineralocorticoid receptor (MR); the other is glucocorticoid receptor (GR; de Kloet et al., 2005). The GR and MR function either as a homodimer or heterodimer, and the affinity of MR for cortical steroids is 10-fold higher than that of GR (de Kloet et al., 2005). The expression of α3, but not α1, in PC12 cells was suppressed in a dose-dependent manner by not only mifepristone (Inoue et al., 2013), a specific GR inhibitor (Reul et al., 1990), but also by RU28318 (unpublished observations by KH and MI), a specific MR inhibitor (Ulmann et al., 1985), suggesting the involvement of MR as well as GR. In fact, the expression of MR in PC12 cells has been reported (Goto et al., 2009). Whether MR is involved in the regulation of α_3_ expression in adrenal chromaffin cells or not remains to be explored. One critical difference between adrenal medulla and brain may be the existence of a higher glucocorticoid level in the extracellular space of adrenal medulla (Wurtman, 2002). Thus, based on the fingdings in PC12 cells, glucocorticoids likely preserve α3 expression in adrenal chromaffin cells. Recently, a decrease in intracellular Cl^−^ concentration ([Cl^−^]_i_) has been shown to be involved in the substitution of α1 for α3 (Succol et al., 2012). The value of [Cl^−^]_i_ in neurons is determined by a balance between Na^+^, K^+^, Cl^−^-cotransporter type 1 (NKCC1) and K^+^, Cl^−^-cotransporter type 2 (KCC2), which are responsible for import and export of Cl^−^, respectively (Kaila et al., 2014). Aldosterone has been reported to enhance NKCC1 activity in vascular smooth muscle cells (Ding et al., 2014) with the consequent increase in [Cl^−^]_i_ (Davis et al., 1993). These results raise the possibility that glucocorticoids enhance NKCC1 activity in chromaffin cells, thereby preserving α3-containing GABA_A_ receptors. A further study will be required to elucidate the molecular mechanism for α3 preservation in chromaffin cells.

## Effects of GABA

### Differences Among Species

The fact that the expression of α3 depends on glucocorticoid activity in PC12 cells indicates that the subunit composition and/or expression of GABA_A_ receptors in cells can be altered in culture. One of the advantages of using acutely dissociated chromaffin cells is that receptors and ion channels in cells can be examined under more physiological conditions compared with cultured cells. The responses of acutely dissociated chromaffin cells to GABA conspicuously differ between rats and guinea pigs. Application of 30 μM GABA evoked inward currents in 33% of rat chromaffin cells examined (Warashina and Inoue, 2012), but in 100% of guinea-pig chromaffin cells (Warashina and Inoue, 2012). In addition, even in responding cells the amplitudes of GABA-induced currents in rat cells were tiny compared with those in guinea-pigs. Such differences, however, did not occur for nicotine-induced currents. Since the dose response relationship for GABA did not differ noticeably between rat (Matsuoka et al., 2008) and guinea-pig chromaffin cells (Inoue et al., 2013), the subunit composition of GABA_A_ receptors in both may be the same, suggesting that the difference in amplitude is the result of different levels of expression. There is a possibility that the difference in the expression level of GABA_A_ receptors is related to the humoral environments in rats and guinea pigs. One difference is that the main glucocorticoid in rats and mice is corticosterone, whereas that in guinea pigs and humans is cortisol. The glucocorticoid activity of corticosterone is one-tenth to one-third that of cortisol (Mahgoub et al., 1997). Therefore, corticosterone may not be sufficient to maintain a high level of expression of α3-containing GABA_A_ receptors in rat adrenal chromaffin cells.

### Dual Action of GABA

The fact that GABA induces excitation in adrenal chromaffin cells with the consequent secretion of catecholamines has been known since the 1980s. As GABA did not change the percentage of adrenaline in the total catecholamine secreted in perfused rat adrenal glands (Inoue et al., 2010), GABA_A_ receptors are likely to be expressed similarly in adrenaline and noradrenaline cells. This non-selectivity is in contrast with the selective expression of M_1_ muscarinic receptors in adrenaline cells, a muscarinic receptor subtype involved in secretion (Inoue et al., 2010; Harada et al., 2015).

**Figure 3:**
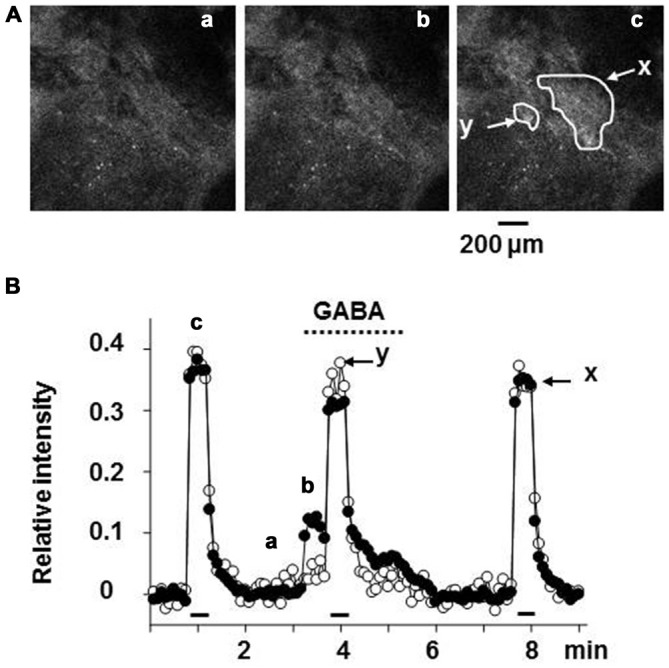
**Suppression of the synaptically evoked Ca^2+^ signal in rat adrenal chromaffin cells by GABA. (A)** Confocal images of Fluo-4 fluorescence in rat adrenal medulla. The adrenal gland was retrogradely loaded with Fluo-4 AM, a Ca^2+^ indicator via the adrenal vein, and then part of the adrenal cortex covering the medulla was removed by microscissors under a stereoscope (Warashina and Inoue, 2012). The adrenal gland was placed between a pair of silver circles for electrical stimulation. Confocal images of FITC-like fluorescence in adrenal chromaffin cells were acquired every 5 s. **(B)** Relative values of the change in fluorescence intensity are plotted against time. The adrenal gland was retrogradely perfused with saline via the adrenal vein, and 30 μM GABA was added to the perfusion solution during the indicated period (dotted line). Nerve fibers were electrically stimulated with 60 V pulses of 1.5 ms duration at 10 Hz for 30 s (bars). Fluorescence intensities in the areas (*x* and *y*) indicated in **(A)** part c were measured and presented as filled (*x*) and open (*y*) symbols, respectively. Note that the cells in the region indicated by *y* showed no response to the application of GABA, whereas those in the region marked x did. After correction for the decline due to photobleaching, the increase in fluorescence intensity was expressed as a fraction of the resting level. The images marked a, b, and c in **(A)** correspond to the points labeled in **(B)**. **(A,B)** Are reproduced from Matsuoka et al. (2008).

The intracellular concentration of Cl^−^ in rat chromaffin cells was electrophysiologically estimated to be 31 mM, as the equilibrium potential for Cl^−^ ([E_Cl_]) is -38 mV (Matsuoka et al., 2008; Inoue et al., 2010). It is likely that the chloride gradient and E_Cl_ in rat chromaffin cells is determined by finely tuned expression and/or function of NKCC1 and KCC2 (Kaila et al., 2014). This value of E_Cl_ may allow GABA to have a dual action; GABA by itself induces excitation, but inhibits the much larger excitation resulting from a volley of neuronal inputs. As shown in Figure 3, GABA alone induced a depolarization with the consequent activation of voltage-dependent Ca^2+^ channels by stimulating GABA_A_ receptors, resulting in catecholamine secretion. When the adrenal medulla was electrically stimulated at a high frequency (5–10 Hz) during GABA_A_ receptor stimulation, the total amplitude of Ca^2+^ signals, which consisted of GABA-induced and synaptically evoked Ca^2+^ responses, was smaller than that evoked synaptically alone (Figure 3). This decrease in the overall Ca^2+^ signal is ascribed to the fact that the membrane electrical shunt induced by GABA (reversal potential at about −38 mV) reduces the depolarization resulting from nerve stimulation and so reduces Ca^2+^ channel activation.

The E_Cl_ in rat chromaffin cells is more negative than the E_Cl_ (−28 mV) in cultured bovine chromaffin cells (Xie et al., 2003). This difference might account for the apparent absence of a shunt effect of GABA_A_ receptor activation in bovine chromaffin cells: the peak amplitude of nicotine-induced Ca^2+^ signals was not altered by GABA_A_ receptor stimulation, even though GABA alone induced an increase in Ca^2+^ concentration (Xie et al., 2003). This failure of GABA to suppress the Ca^2+^ signal may be due to the E_Cl_ of −28 mV, which is likely enough to activate a substantial number of voltage-dependent Ca^2+^ channels. This more positive value of E_Cl_ in bovine chromaffin cells might be due to the maintenance of cells under culture conditions for a few days, which may preferentially facilitate the expression of NKCC1 rather than KCC2 with the consequent increase in [Cl^−^]_i_.

A possible dual action of GABA in adrenal chromaffin cells could have a significant physiological function. When the live organism is subjected to a stressor, the organism would respond to it with a rapid, nerve evoked secretion of catecholamines from the adrenal medulla, which results in increases in blood glucose and blood flow in the skeletal muscle. However, if a high glucose level is sustained, it would be harmful to the body, resulting in the outbreak or exacerbation of the metabolic syndrome. On the other hand, a low level of blood adrenaline would be beneficial in that it ameliorates the metabolic syndrome by facilitating glucose uptake with the consequent decrease in blood glucose (Ziegler et al., 2012). In this manner, the dual action of GABA might both enhance low levels of release and dampen high levels, and so would contribute to a delicate regulation of catecholamine secretion.

## Actions of Neuroactive Steroids

**Figure 4:**
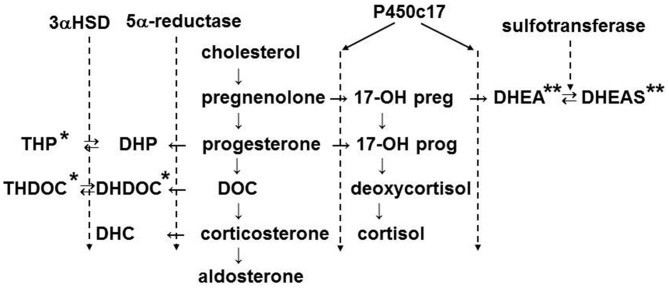
**Biosynthetic pathway of adrenal steroids.** The enzymes shown mediate the conversion of steroids in the indicated step(s). The names of steroids are abbreviated: THP, 3α, 5α-tetrahydroprogesterone or allopregnanolone; DHP, 5α-dihydroprogesterone; THDOC, 3α, 5α-tetrahydrodeoxycorticosterone; DHDOC, 5α-dehydrodeoxycorticosterone; DHC, dehydrocorticosterone; DOC, 11-deoxycorticosterone; 17-OH preg, 17-OH pregnenolone; 17-OH prog, 17-OH progesterone; DHEA, dehydroepiandrosterone; DHEAS, DHEA sulfate. 3αHSD, 3α-hydroxysteroid dehydrogenase. The steroids marked by single and double asterisks are positive and negative allosteric modulators of GABA_A_ receptors, respectively.

Neuroactive steroids, such as allopregnanolone, are produced in adrenal glands of mammals including rats (Corpéchot et al., 1993) and humans (Genazzani et al., 1998) and are known to act on GABA_A_ receptors (Zhu and Vicini, 1997; Belelli and Lambert, 2005). Therefore, to elucidate the function of GABA signaling in the adrenal medulla, it will be important to take into account the effects of neuroactive steroids. Figure 4 illustrates the biosynthetic pathway for adrenal steroids, in which positive (Lambert et al., 1995; Reddy and Rogawski, 2002) and negative (Park-Chung et al., 1999) allosteric modulators are denoted by single and double asterisks, respectively. The adrenal cortex of rats and mice lacks the expression of P450c17 (Le Goascogne et al., 1991; Figure 4). Therefore, the main glucocorticoid in such rodents is corticosterone, whereas in other mammals including humans it is cortisol. The lack of P450c17 results in a loss in production of dehydroepiandrosterone (DHEA) and its sulfated form or DHEAS in the adrenal cortex. In rat gonads where P450c17 is expressed, DHEAS is produced. In contrast, allopregnanolone (THP in Figure 4) is produced in the adrenal glands of rats (Corpéchot et al., 1993) and humans (Genazzani et al., 1998). It is synthesized in two steps from progesterone. 5α-reductase, which was immunologically detected in the zona fasciculata of rat adrenal glands (Yokoi et al., 1998), catalyzes the conversion from progesterone to 3α-hydroprogesterone (DHP), which is further converted to allopregnanolone by 3α-hydrosterone dehydrogenase.

### Allopregnanolone

**Figure 5:**
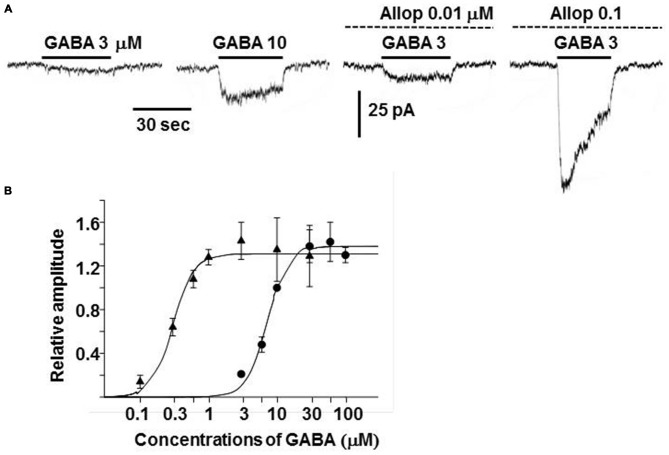
**Enhancement of GABA_A_ receptor channel activity by allopregnanolone. (A)** Whole-cell currents recorded from a dissociated guinea-pig chromaffin cell at −60 mV. The traces were obtained from the same cell. GABA at 3 or 10 μM was bath applied during the indicated period (bars) in the absence and presence of 0.01 or 0.1 μM allopregnanolone (interrupted lines). **(B)** Dose-response curves for GABA-induced currents at the plateau level in the absence (•) and presence (▴) of 0.1 μM allopregnanolone. The lines show fits of logistic equations with I_Max_s of 1.38 and 1.31, slope factors of 2.88 and 2.83, and EC_50_s of 7.2 and 0.3 μM in the absence and presence of allopregnanolone, respectively. Plateau amplitudes of GABA-induced currents are expressed as fractions of those of 10 μM GABA currents in the same cells. The data represent mean ± SEM. **(A,B)** Are reproduced from Inoue et al. (2013).

Exposure of guinea-pig adrenal chromaffin cells to allopregnanolone resulted in an enhancement of GABA_A_ receptor channel activity (Inoue et al., 2013). In the presence of 0.01 and 0.1 μM allopregnanolone, the amplitude of currents induced by 3 μM GABA increased by 1.1 and 20-fold (Figure 5A). In addition, the dose-response curve for steady-state GABA-induced currents was shifted toward the left and the EC_50_ decreased by 24-fold in 0.1 μM allopregnanolone (from 7.2 μM to 0.3 μM; Figure 5B). This result indicates that 0.1 μM GABA, which does not induce any channel activity in the absence of allopregnanolone, is able to produce a sustained current in the presence of 0.1 μM allopregnanolone. The blood concentration of GABA in humans is about 0.1 μM (Petty, 1994), but the concentration of GABA in the extracellular space of the adrenal medulla is not known. Since GABA is thought to be released from chromaffin cells (Kataoka et al., 1984), the concentration of GABA in the vicinity of chromaffin cells is expected to be larger than the 0.1 μM concentration observed in the blood. The plasma concentration of allopregnanolone in rats has been reported to be a few nanomolar levels at rest and to be elevated to 20 nM in stress, such as swimming (Purdy et al., 1991). However, the adrenal vein drains the venous blood from the adrenal cortex through the medulla and so it is very likely that the concentration of allopregnanolone (synthesized in the cortex) will be elevated over that in the general circulation. In light of the concentrations at which the GABA_A_ receptor channel activity in chromaffin cells is enhanced, allopregnanolone may play an important role in modulating the para/autocrine function of GABA in adrenal medullae.

### DHEAS

As explained above, DHEAS is not produced in the rat adrenal cortex and its plasma concentration is at nanomolar levels (Cutler et al., 1978). On the other hand, it is a major steroid in the human adrenal cortex and its plasma concentration is 1–10 μM (Dhatariya and Nair, 2003). Therefore, DHEAS possibly has a modulatory effect on GABA signaling in the adrenal medulla of at least humans. An electrophysiological analysis of the effects of DHEAS on GABA_A_ receptor channels in rat pituitary glands revealed that DHEAS inhibits channel activity by acting at two different sites: one is a high apparent affinity of 0.2 nM and the other is a low apparent affinity of 15 μM (Hansen et al., 1999). In guinea-pig chromaffin cells, bath application of DHEAS inhibited GABA_A_ receptor channels in a reversible manner (unpublished observations by MI). The dose-dependent inhibition by DHEAS was well fitted with an inhibition curve with an IC_50_ of 3 μM and a Hill’s coefficient of 1. This low potency of inhibition would negate the possible regulation of GABA signaling in the rat adrenal medulla by DHEAS. However, it might be involved in the regulation of GABA function in the human adrenal medulla.

## Concluding Remarks and Perspective

In many respects GABAergic transmission in the rat adrenal medulla resembles that in the developing brain. GABA is believed to be involved in the maturation process of the brain in the embryonic period, including proliferation of neurons, their trafficking, and synaptogenesis (Represa and Ben-Ari, 2005). These effects of GABA are mediated by GABA_A_ receptor-mediated depolarization and the subsequent activation of voltage-dependent Ca^2+^ channels. GABA_A_ receptor α subunits are divided into immature (α2 and α3) and mature (α1) types; the α2 and α3 subunits are predominantly expressed in the embryonic brain and the α3 in adult chromaffin cells. A similar distinction is noted in GADs involved in GABA synthesis. GAD67 (immature isoform) is expressed at an early stage in brain development and in adult chromaffin cells, while GAD65 (mature isoform) expression increases in the brain during synaptogenesis. GAD65 is physically associated via palmitoylation with synaptic vesicles in the nerve terminal (Kanaani et al., 2004), whereas GAD67 is diffusely present not only in the nerve terminal, but also in the cell body. It has been suggested that in the embryonic brain GABAergic transmission, which occurs in vesicular and non-vesicular manners (Cellot and Cherubini, 2013), functions in a more general, volume transmission mode that converts to a more precise, synaptic mode with development (Represa and Ben-Ari, 2005). In adult chromaffin cells there is no evidence for GABAergic synapses, and the isoforms for GAD and the GABA_A_ receptor α subunit expressed in chromaffin cells are the immature forms. Finally, the lack of expression of plasma membrane GABA transporters (GATs; Matsuoka et al., 2008) in the adrenal gland, which are expressed in nerve terminals and glia to remove GABA from the synaptic cleft in the brain (Dalby, 2003) would enhance the ability of GABA to spread through the extracellular space. These considerations suggest that in the adrenal medulla GABA plays a para/autocrine role: it is released by chromaffin cells and acts on the same or neighboring cells to modulate catecholamine release. At least in the rat adrenal gland GABA plays a dual role. When GABA alone is applied it results in release of catecholamines. However, due to the membrane shunting effect of the chloride conductance, GABA also serves to reduce the large release elicited by volleys of nerve impulses.

**Figure 6:**
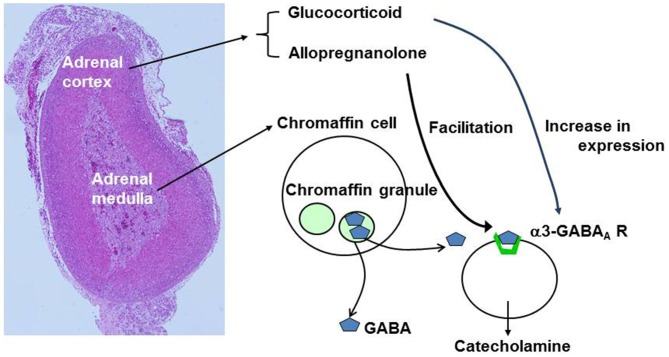
**Summary of effects of adrenal cortical hormones on GABA signaling.** The left image represents hematoxylin-eosin staining of mouse adrenal gland. GABA is stored in large dense core vesicles (LDCVs) or chromaffin granules in chromaffn cells. Glucocorticoids produce an increase in expression of α3-containing GABA_A_ receptors whereas allopregnanolone produces facilitation of GABA_A_ receptor Cl^−^ channel activity.

GABAergic modulation of catecholamine release is also under a second level of control by adrenal cortical cells (Figure 6). The intra-adrenal portal vascular system (Coupland, 1975) drains the cortex that secretes steroid hormones including glucocorticoids and allopregnanolone. The portal system then enters the medulla, where chromaffin cells will be exposed to the secreted hormones at high concentrations (Wurtman, 2002). Hence, increased steroid release in response to stress or other stimuli will affect GABAergic modulation of catecholamine release; glucocorticoids by possibly enhancing the expression of GABA_A_ α3 subunit and allopregnanolone by directly potentiating responses of GABA_A_ receptor to low concentrations of GABA. Both of these actions would enhance the ability of low levels of GABA to induce release of catecholamines. However, they would also increase the ability of GABA to reduce release induced by high frequency nerve stimulation. These effects would integrate activation of the cortical cells (perhaps reflecting stress levels) with rapid effects of released catecholamines.

The next challenge is to elucidate how the function and expression of these GABA signaling molecules are regulated to accomplish the para/autocrine function of GABA in adrenal chromaffin cells. Since adrenal chromaffin cells are exposed to adrenal cortical hormones at high concentrations through the intra-adrenal portal vascular system (Coupland, 1975), adrenal cortical hormones, such as glucocorticoids, are a first target to be investigated for their role involved in regulation and/or expression of the signaling molecules.

## Author Contributions

MI, KH, and YY had conception of research; KH, HM, HF, YU, YY, and MI performed experiments; KH and MI analyzed data; MI wrote the manuscript; All the authors approved the final version of the manuscript.

## Conflict of Interest Statement

The authors declare that the research was conducted in the absence of any commercial or financial relationships that could be construed as a potential conflict of interest.
